# The soluble suppression of tumorigenicity 2 as a biomarker of early cardiac remodeling in bradycardia patients receiving permanent pacemaker therapy

**DOI:** 10.2144/fsoa-2023-0001

**Published:** 2023-03-03

**Authors:** Mohammad Iqbal, Iwan Cahyo Santosa Putra, Rizki Bunawan, Hanna Goenawan, Mohammad Rizki Akbar, Arief Sjamsulaksan Kartasasmita, Young Hoon Kim

**Affiliations:** 1 Department of Cardiology & Vascular Medicine, Faculty of Medicine University of Padjadjaran, Bandung, 40161, Indonesia; 2 Department of Internal Medicine, Division of Cardiology, Korea University Medical Center, 73 Inchon-Ro, Seongbuk-Gu, Seoul, 02841, Republic of Korea; 3 Department of Biomedical Sciences, Division of Physiology, Faculty of Medicine University of Padjadjaran, Bandung, 45363, Indonesia; 4 Faculty of Medicine University of Padjadjaran, Bandung, 45363, Indonesia

**Keywords:** early cardiac remodeling, permanent pacemaker, right ventricular pacing, soluble suppression tumorigenicity-2 level

## Abstract

**Aim:**

This study aims to evaluate: the difference of soluble suppression of tumorigenicity 2 (sST2) level, a biomarker for cardiac remodeling and echocardiography parameters value prior to and 1 month after implantation; and the association between pacemaker parameters and pacemaker mode along with delta sST2 levels.

**Materials & methods:**

This prospective cohort study enrolled all symptomatic bradycardia patients aged >18 years with preserved ejection fraction who underwent permanent pacemaker (PPM) implantation.

**Results:**

A total of 49 patients were included in this study. The sST2 level (ng/ml) were significantly different between prior and 1 month following PPM implantation (23.4 ± 28.4 vs 39.9 ± 63.7; p = 0.001).

**Conclusion:**

The early cardiac remodeling has occurred within 1 month after PPM implantation as indicated by increasing delta sST2 level.

## Plain language summary

It is widely known that pacing induced cardiomyopathy, which results from the utilization of a permanent pacemaker (PPM) within a long-term duration, will increase the risk of mortality and morbidity. Hence, early detection of the cardiac remodeling process is warranted in order to prevent this course. In this study, the soluble suppression of tumorigenicity 2 level (ng/ml), known as an indicator of cardiac remodeling, was significantly higher in 1 month following PPM implantation compared with the baseline (23.4 ± 28.4 vs 39.9 ± 63.7; p = 0.001). Thus, it denotes that early cardiac remodeling might occur earlier than expected, within 1 month following PPM implantation.

According to the most recent guideline, permanent pacemaker (PPM) with right ventricular (RV) pacing is still a mainstay therapy of bradycardia in patients with preserved ejection fraction (EF) [1]. However, RV pacing in the long-term duration can lead to ventricular desynchronization, thereby increasing the risk of pacing induced cardiomyopathy (PICM) within months up to years [2,3]. Hence, researcher has found several factors that may decrease the risk of PICM including the right ventricular outflow tract (RVOT) pacing, low paced QRS duration and low pacing percentage [2,4–7].

Although a long-term process is necessarily required to cause PICM, the early cardiac remodeling might occur within 1 month after PPM placement [8,9]. The term cardiac remodeling refers to a series of molecular, cellular and interstitial changes that are clinically manifested by changes in the size, mass, geometry and function of the heart following a cardiac injury [10]. Accordingly, an observational study by Xu *et al.* showed that gene expression which regulates cardiomyocytes metabolism was altered within 1 month after PPM implantation [8]. Moreover, subclinical LV systolic dysfunction, reflecting by decline of global longitudinal strain was occurred within 1 month after implantation [9]. However, it still equivocal whether the early cardiac remodeling due to PPM implantation is occurred in such short duration of time.

Based on the current guideline, the soluble suppression of tumorigenicity 2 (sST2) level measurement was recommended as a prognostic marker of heart failure (HF), which increased of this biomarker’s level was correlated with several detrimental outcomes [11]. Nonetheless, the utilization of this biomarker’s has grown significantly as it can also detect the cardiac remodeling process [12,13]. To the best of our knowledge, there are no study that evaluated the sST2 level in order to detect early cardiac remodeling in bradycardia patients undergoing PPM implantation. Hence, this prospective cohort study aims to evaluate: the difference of sST2 level and echocardiographic parameters value prior and 1 month after PPM implantation; and the association between pacing parameters (pacing location, pacing percentage and paced QRS duration) and pacing mode along with delta sST2 level.

## Materials & methods

### Study design & patient selection

This is a single-center prospective cohort study that enrolled all symptomatic bradycardia patients aged >18 years. Patients who underwent PPM implantation due to symptomatic bradycardia and had left ventricular ejection fraction (LVEF) prior implantation higher than 50% were included. Patients who presented with acute coronary syndrome, acute or chronic HF, valvular heart disease, congenital heart disease, congenital cardiac conduction abnormalities, poor echocardiography window, cardiomyopathy (e.g., dilatation, restrictive or hypertrophic), myocarditis, infection disease, autoimmune disease, acute asthma exacerbation and loss of follow-up were excluded from this study.

### Definition & variable outcome

A decision of choosing PPM mode, either single or dual chamber (VVI or DDD) was made by physician according to current guidelines [1]. We implanted VVI in patient with sinus node dysfunction or AV block if the patient has any reason to avoid two leads or has significant comorbidity as recommended by current guidelines [1]. Patients who were implanted by single chamber with mode AAI were not included in this study. The lower rate of VVI was determined based on baseline patient heart rate. For example, when the patient had a sinus node disease (with intermittent sinus pause), and the baseline heart rate was about 55–60 bpm, the lower rate was set at 50 bpm. The sleep rate or rest rate was set up case by case based on physician decision. This setting was important to avoid unnecessary RV pacing. In dual-chamber PPM, the atrioventricular (AV) interval was set up in the default setting (paced AV interval was 150 ms and sensed AV interval 120 ms). Meanwhile, in case of the prolonged PR interval, AV interval was set up (max of 250 ms) according to the physician’s decision to reduced unnecessary RV pacing.

All PPM implantation was carried out by an experienced operator. Independent variables of the current study are collected (pacing mode, pacing location, pacing percentage and paced QRS duration). In this study, we only performed PPM implantation in two different locations such as RVOT pacing and RV apical pacing. The RVOT pacing was preferable rather than RV apical pacing whenever possible, unless the parameters RVOT pacing (such as impedance, threshold, etc.) was not satisfactory. High septal pacing was classified as RVOT pacing in this study. Either RVOT pacing or RV apical pacing was achieved with an active fixation lead. The appropriate positioning of the lead was fluoroscopically confirmed at the time of implantation and by the QRS axis (during pacing) from ECG which was recorded after implantation. Right ventricle apical pacing was considered when the QRS axis in inferior leads are predominantly negative, and RVOT pacing were considered when QRS axis in inferior leads of ECG are predominantly positive. The pacing burden (pacing percentage) and other PPM parameters were determined from stored data after 1 month of implantation. A standard 12 lead surface ECG was obtained at speed 25 mm/s. The paced QRS duration was defined as the length of time from the beginning of the pacing artifact (spike) to the end of the QRS complex during RV pacing. The QRS duration was measured using a digital calliper (KW06-351, Krisbow).

The sST2 were collected prior and after 1 month of implantation. In regard to measure sST2 level, a total of 3 cc of blood sample was taken from each included patient. Blood samples were collected in tubes containing EDTA. Samples were centrifuged for 15 min at 4°C and stored at 2–8°C. Level of sST2 were measured using the Presage^®^ ST2 Assay according to manufacturer instructions. The delta sST2 is a comparison level of sST2 prior and 1 month after implantation. While the outcome of this study is delta sST2 which described as difference level of sST2 1 month following implantation and 1 week prior implantation.

### Statistical analysis

All statistical analyses were performed using SPSS, version 25.0 (SPSS, Inc, IL, USA). The Shapiro–Wilk test was used to assess data distribution (normality data). All numerical data were expressed as means ± standard deviations. Whereas categorical data were summarized as frequencies and percentages. Univariate analysis was performed wielding unpaired Student’s *t*-test or Mann–Whitney U test to calculate the association between two numerical data. Furthermore, in order to compare the sST2 level, QRS duration and echocardiographic parameters prior and after implantation, paired Student’s *t*-test or Wilcoxon test were used. Student’s *t*-test was preferred to use if distribution data were normal, whereas Mann–Whitney U and Wilcoxon tests were obtained when distribution data was not normal. Univariate and multivariate analysis were performed using linear regression analysis to discover which variables who modified the sST2 level after implantation. All probability values were deemed statistically significant at a level of <0.05.

## Results

### Patient selection

**Figure 1.**
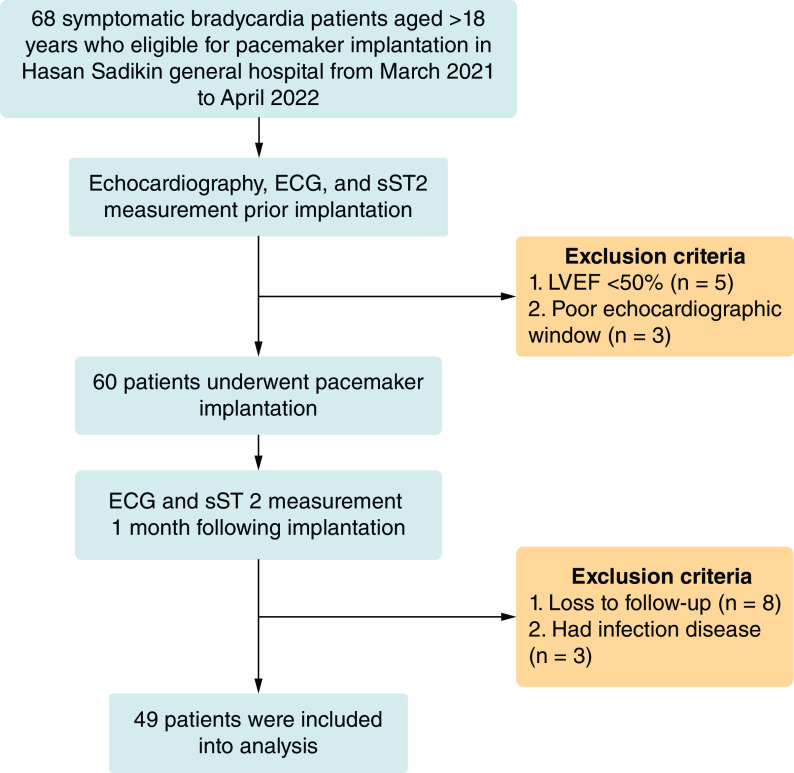
Patients selection process. ECG: Electrocardiography; LVEF: Left ventricular ejection fraction; sST2: Soluble ST2.

For starters, there are 68 symptomatic bradycardia patients aged >18 years who underwent PPM implantation in the beginning of study. Moreover, 19 of them were excluded due to LVEF <50% (n = 5), poor echocardiography window (n = 3), loss to follow-up (n = 8) and suffer infection disease (n = 3), subsequently 49 patients were finally included into analysis. Patients’ selection process is described in Figure 1.

### Baseline characteristics of included participants

Most of the included patients were elderly (66.5 ± 11.5 years), male (57.1%), and non-smokers (65.3%). Hypertension (67.3%) was the most common comorbidities among included patients, following by dyslipidemia (51%), coronary artery disease (44.9%) and diabetes mellitus (26.5%). Concerning prior medication, the majority of patients received statin (57.1%), calcium channel blocker (46.9%) and antiplatelet (44.9%), whereas only 8.1% of patients took ACEI/ARB and 10.2% consumed beta-blocker.

Among included patients, sinus node dysfunction was the main etiology of bradycardia, accounts to 59.2%. Dual chamber pacing (63.3%) was commonly performed in this study. The location of PPM was comparable between in RV apical (44.9%) and RVOT pacing (55.1%). Regarding ECG and echocardiography results, the mean of QRS duration and LVEF appears normal. Moreover, pacing percentage in our study showed a high burden pacing (1 month following implantation). Baseline characteristics are elaborated in Table 1.

**Table 1.** Baseline characteristics of included participants.

Variables	n = 49
Age (years), mean ± SD	66.5 ± 11.5
Sex Male, n (%) Female, n (%) Smokers, n (%)	28 (57.1) 21 (42.9) 17 (34.7)
Comorbid Coronary artery disease, n (%) Diabetes mellitus, n (%) Hypertension, n (%) Dyslipidemia, n (%)	22 (44.9) 13 (26.5) 33 (67.3) 25 (51)
Medication used, n (%) Statin Calcium channel blocker ACEI/ARB Antiplatelet Beta blocker	28 (57.1) 23 (46.9) 4 (8.1) 22 (44.9) 5 (10.2)
Bradycardia etiologies Sinus node dysfunction, n (%) Atrioventricular block, n (%)	29 (59.2) 20 (40.8)
PPM pacing location Right ventricular apex, n (%) RVOT, n (%)	22 (44.9) 27 (55.1)
QRS duration (s) Prior implantation, mean ± SD	0.097 ± 0.02
Echocardiographic parameter LVEF prior implantation – biplane Simpson’s (%) Pacing (cumulative) percentage (%)	63.8 ± 6.1 63.2 ± 35.6

All categorical variables are expressed by n (%) and all numerical variables are expressed by mean ± SD.

ACEi: Angiotensin converting enzyme inhibitor; ARB: Angiotensin receptor blocker; LVEF: Left ventricular ejection fraction; PPM: Permanent pacemaker; PPM: Permanent pacemaker; RVOT: Right ventricular outflow tract; SD: Standard deviation.

### The comparison of sST2 levels, echocardiography results, QRS duration prior & after implantation

The univariate analysis showed that sST2 level 1 month following implantation was significantly higher compared with sST2 level prior implantation (p = 0.001) (Table 2). The statistical analysis showed that LVEF, left ventricle internal diameter at diastolic and left ventricle internal diameter at systolic remains unchanged (p > 0.05). However, the QRS duration during pacing was significantly different compared with the baseline (<0.001) (Table 2).

**Table 2.** Soluble suppression of tumorigenicity 2 levels, QRS duration and echocardiographic parameters before and after implantation.

Variable	Baseline (n = 37)	1 month after implantation (n = 37)	p-value
sST2 level (ng/ml)	23.4 ± 28.4	39.9 ± 63.7	0.001
QRS duration (s)	0.097 + 0.02	0.14 + 0.03	<0.001
Echocardiographic parameters			
LVEF (%)	63.8 ± 6.1	62.71 ± 5.9	0.072
LVIDd (millimeter)	46.4 ± 5.06	47.2 ± 5.48	0.368
LVIDs (millimeter)	29.4 ± 7.8	32.05 ± 8.6	0.093

LVEF: Left ventricle ejection fraction; LVIDd: Left ventricle internal diameter at diastolic; LVISd: Left ventricle internal diameter at systolic; sST2: soluble suppression of tumorigenicity 2.

### The association between pacemaker & electrocardiography parameters along with delta sST2

The analysis revealed that delta sST2 was not significantly changed regardless of pacing location (p = 0.117) and pacing mode (p = 0.330) (Table 3). Univariate linear regression analysis showed that pacing cumulative percentage (p = 0.100) and QRS duration after implantation (p = 0.646) were not remarkably associated with delta sST2 (Table 4).

**Table 3.** Comparison of delta soluble suppression of tumorigenicity 2 level based on pacing location and pacing mode.

Variable pacing location	Delta sST2	p-value
Pacing location		
RV apical	15.2 ± 25.05	0.117
RVOT	17.9 ± 61.8	
Pacing mode		
VVI	42.3 ± 72.1	0.330
DDD	35.7 ± 47.5	

All numerical variable was presented as mean ± SD.

DDD: Dual chamber ventricular pacemaker; sST2: Soluble suppression of tumorigenicity 2; RV: Right ventricle; RVOT: Right ventricular outflow tract; VVI: Single chamber ventricular pacemaker.

**Table 4.** Univariate linear regression analysis of association between pacing and electrocardiography parameters along with delta soluble suppression of tumorigenicity 2.

Variable	Univariate			
	B	SE	95% CI	p-value
Pacing percentage	-0.3	0.179	-0.66; 0.06	0.100
Paced QRS duration	-116.3	251.7	-662.7; 390.1	0.646

B: Coefficient B; SE: Standard error; sST2: Soluble suppression of tumorigenicity 2.

Based on multivariate linear regression, these three pacing parameters including pacing location (p = 0.808), pacing cumulative percentage (p = 0.079), QRS duration after implantation (p = 0.348) and pacing mode (single vs dual chamber) (p = 0.887) were not substantially correlated with delta sST2 (Table 5).

**Table 5.** Multivariate linear regression analysis of association between pacing and electrocardiography parameters along with delta soluble suppression of tumorigenicity 2.

Variable	Multivariate			
	B	SE	95% CI	p-value
Pacing location	3.230	14.44	-25.9; 32.33	0.824
Pacing percentage	-0.338	0.191	-0.72; 0.05	0.084
Paced QRS duration	-266.9	281.9	-835.2; 301.2	0.349
Pacing mode	-1.953	13.67	-29.51; 25.6	0.887

B: Coefficient B; SE: Standard error; sST2: Soluble suppression of tumorigenicity 2.

## Discussion

To the best of our knowledge, this is the first study to detect early cardiac remodelling by sST2 measurement in patient who underwent PPM implantation. This study yielded three main results. For starter, sST2 level was significantly increased within 1 month after PPM implantation, indicating that early cardiac remodeling process was occurred. Second, echocardiographic parameters including LVEF, left ventricle internal diameter at diastolic and left ventricle internal diameter at systolic remained unchanged after 1 month following pacing placement (Table 2), denoting that cardiac remodeling was initiated although the structure and function of the heart were still normal. In another point of view, it may conclude that echocardiographic evaluation was less sensitive compared with sST2 measurement in terms of detecting early cardiac remodeling in patients with PPM. Third, pacing parameters including pacing location, pacing percentage, and paced QRS duration were not substantially associated with delta sST2 level. It implied that early cardiac remodeling process after PPM placement was inevitable. According to our results, we suggest the utilization of sST2 as a predictor of early cardiac remodeling in bradycardia patients with PPM.

It is generally known that long-term complications of prolong RV pacing is PICM, due to its capability to cause electrical dyssynchrony. The electrical dyssynchrony will triggered mechanical stretch and biomechanical stress and it will trigger cardiac remodeling cascade just soon after the pacing [2,3]. Xu *et al.* study found that RV pacing (especially apical pacing) may alter gene expression Optic Atrophy 1 (OPA1) and Sarcoplasmic Reticulum Calcium ATPase2a (SERCA2a) within 1 month after pacing placement. These genes regulate cardiac mitochondrial energy metabolism and cardiac contractile function [8]. Early changes in expression of OPA1 and SERCA2a were associated with deterioration of LVEF that became apparent months later [8]. Another study showed that the decline of GLS has occurred within 1 month after PPM implantation, despite no change of LVEF [9]. Therefore, the significant cardiac remodeling may occur earlier than expected. Moreover, our study also supports this theory by showing that early cardiac remodeling which indicated by increasing sST2 level remarkably, was initiated within 1 month after RV pacing.

The sST2 has been known as a cardiac remodelling marker since 2002 [13]. The secretion of sST2 was initiated by mechanical stretch and biomechanical stress within cardiac muscle [14]. Thus, this marker was associated with cardiomyocyte remodeling, as it can detect the myocardial hypertrophy and myocardial fibrosis [12]. However, unfortunately, fixed and validated cut-off of this biomarker in identifying cardiac remodeling is not available yet. It is due to diversity of population characteristics, difference of sST2 assay, and variety of HF etiologies [15]. Thus, in order to enhance the clinical implications of sST2 analysis in bradycardia patients with PPM, the validation and determination of fixed cut-off point is warranted.

The association between several pacing parameters including pacing sites, pacing percentage and paced QRS duration along with PICM have been described by several previous studies. Gong *et al.* showed that 12 months of RVOT pacing in patients with normal cardiac function has no benefit in term of preventing cardiac remodeling and preserving LV systolic function, despite the fact that RVOT pacing presented more synchronous LV contraction compared with RV apical pacing [4]. Furthermore, an observational study by Sweeney *et al.* have demonstrated that in patients with preserved LVEF, the risk of HF after RV apical pacing was low [3] Similarly, another study by Chiladakis *et al.* also suggested that RV apical pacing provided no harmful effects to patients with normal LV systolic function [16]. On the other hand, Victor *et al.* study identified that RV septal pacing may offer more beneficial outcomes as opposed to RV apical pacing but only in patient with baseline LVEF <45%, and not in patients with LVEF >45% [5]. Our study showed that there was no difference of delta sST2 between RV apical pacing group compared with RVOT pacing group within 1 month after implantation. All of our subjects have preserved LVEF. Therefore, we concluded that the pacing sites (RV apical pacing and RVOT pacing) may be less importance to see the difference of sST2 in patient with preserved LVEF, or it may less importance in short term period. The difference of delta sST2 between these two groups might be difference in long term of follow-up.

Similar to pacing sites, numerous studies have described that number of pacing percentage may also predict the incidence of PICM in the future. According to Sweeney *et al.* study, it showed that pacing percentage >40% have a higher risk to develop PICM, regardless of the PPM mode (single or dual chambers) [17] Moreover, Zhang *et al.* [18] showed that pacing percentage >90% may prognosticate the PICM incidence, while Kiehl *et al.* [2] suggested a lower cut off of pacing percentage which is >20% to defined patients who had higher risk of PICM. However, up until now, neither consensus nor guideline describe the validated cut off of pacing percentage that ideally used to predict PICM. Our study showed that pacing percentage was not correlated with delta sST2 level despite of high burden pacing (pacing percentage) in our subjects (63.2 + 35.5). Therefore, the relationship between pacing percentage and delta sST2 might be seen in study with long term of follow-up and larger sample size.

Given to the paced QRS duration were directly indicated an electrical dyssynchrony, this parameter has been evaluated by several studies for its potential to predict the risk of PICM [19]. A cohort study conducted by Sweeney *et al.* showed that wider paced QRS duration was associated with worsening LV systolic function and increase the risk of HF in patients with pacemaker [20]. Accordingly, Kim *et al.* study has demonstrated that paced QRS duration >140 ms and >167 ms had sensitivity and specificity of 95 and 90%, respectively, to prognosticate the incidence of PICM [7]. Furthermore, Khursid *et al.* and Miyoshi *et al.* pointed >150 and >190 ms, respectively, as ideal cutoffs for predicting PICM [6,21]. However, similar to pacing percentage, the fixed cut-off of paced QRS duration to predict PICM has not been widely validated. Based on our finding, the QRS duration has significantly changed between prior and after implantation (Table 2). However, further analysis showed that paced QRS duration was not linked to delta sST2 within 1 month following PPM placement. If we compared the paced QRS duration in our participants to the previous studies conducted by Kim *et al.* [7], Khursid *et al.* [6] and Miyoshi *et al.* [21], the paced QRS duration in our study was relatively narrow. Therefore, the electrical dyssynchrony in our subject might be less and inferior compared with the previous study, thus it may explain why the paced QRS duration in our study was not correlated with delta sST2 level. Furthermore, the short term follow-up may also play an important role to delineate why paced QRS duration was not associated with delta sST2 in our study.

Based on two cohort studies, pacemaker mode (single chamber VVI and dual chamber DDD) was not significantly correlated with PICM incidence [22,23]. Similarly, our study also found that neither single chamber nor dual chamber PPM were associated with delta sST2 levels, referring that AV synchrony did not delay or prevent early cardiac remodeling process. Also, it may explain why pacemaker mode was not significantly associated with poor prognostic outcome and mortality, event in patient with complete heart block [1].

Since none of these pacemaker parameters and pacemaker mode were not associated with delta sST2 level, we speculated that the mechanism of electrical dyssynchrony due to RV pacing is more complicated. The pacing parameters (pacing sites, pacing percentage, paced QRS duration and pacing mode) and AV synchrony may be not the only pathway which triggered the cardiac remodeling in RV pacing. Multiple factors may be involved in the progression of cardiac remodeling due to RV pacing, such as myocardial necrosis, cardiomyocytes traction, mechanical stress, apoptosis, autophagy, fibrosis, oxidative stress, inflammatory response and neurohumoral regulatory disorders [24,25].

The current guidelines still recommended the utilization of conventional pacemaker in symptomatic bradycardia patient with preserved LVEF [1]. However, according to our findings, we believed that a significant elevation of sST2 level after conventional PPM implantation followed by none of included patients had HF related symptoms and had any alteration of cardiac structure and function based on echocardiography test, indicating that these patients already had subclinical HF which fulfilled the stage A or B HF criteria according to the HF guidelines [26]. Hence, in order to achieve an early diagnosis of HF, we highly suggest that all patients who implanted the PPM to routinely visit a physician, especially those who had significant elevation of sST2 level in the short-term period. At last, our study raises two important questions: whether anti remodeling drugs should be initiated early to prevent or delay risk of PICM, despite of patient has preserved LVEF; and whether we should abandon the conventional pacemaker in the future and perform the physiological pacing, such as his bundle pacing or conduction system pacing.

This study has three main limitations. First, this study did not provide several laboratory data, including HbA1c and inflammation markers (IL-6, c reactive protein [CRP] and procalcitonin) that could affect the sST2 level, indicating an increased risk of bias. However, in the beginning of the study, we have excluded patient with inflammatory diseases, including myocarditis, infection disease and autoimmune disease, presupposing that included patients have normal inflammation markers. Second, this study did not collect other cardiac remodeling markers, including N-terminal pro-brain natriuretic peptide, galectin-3, GDF-15 and syndecan-1. Hence, the indicator of early cardiac remodeling is solely dependent on significant changes in sST2 levels. Hence, we highly suggest other cohort studies to evaluate the usefulness of other cardiac remodeling markers as an indicator of early cardiac remodeling in patients who underwent PPM therapy in order to support our hypothesis that early cardiac remodeling is initiated in short-term period after the procedure. Third, this study had short-term follow-ups within only 1 month and low sample size; thereby, it may address why several associations (especially pacing parameters) in our analysis were insignificant. Therefore, several prospective cohort studies with a long-term follow-up period and larger sample size are needed to evaluate better the comparison of sST2 level and echocardiographic markers before and after implantation to confirm the occurrence of the early cardiac remodeling process, as well as the association between pacing parameters to delta sST2.

## Conclusion

In conclusion, this prospective cohort study revealed that early cardiac remodeling has occurred within 1 month after PPM implantation as indicated by increasing delta sST2 level significantly, even though the cardiac structure and function remains unchanged as shown by echocardiographic parameters. However, the pacemaker parameters including pacing sites, pacing percentage, paced QRS duration and pacemaker mode were not associated with early cardiac remodeling. Thus, several prospective cohort studies with long-term follow-up duration and large sample size are required to confirm our findings.

**Summary points**

- Application of a permanent pacemaker (PPM) in bradycardia patients within a long-term period is associated with pacing induced cardiomyopathy.
- This is the first prospective cohort study that perform early detection of the cardiac remodeling process by measuring the soluble suppression of tumorigenicity 2 (sST2) level; thereby, it will enhance our prevention strategy in intercepting this progressive course.
- This study revealed that sST2 levels were significantly higher after 1 month following PPM implantation compared with the baseline, indicating that early cardiac remodeling occurred.
- Echocardiographic parameters, including left ventricular ejection fraction, left ventricle internal diameter at diastolic and left ventricle internal diameter at systolic, remained unchanged after 1 month following pacing placement, denoting that early cardiac remodeling was initiated despite of normal structure and function of the heart.
- Pacing parameters such as pacing location, pacing percentage, and paced QRS duration were not significantly linked with delta sST2 level, implying that the early cardiac remodeling process after PPM was inevitable.
- According to this study’s findings, we suggest that utilization of sST2 is needed to predict early cardiac remodeling in bradycardia patients undergoing PPM.
